# Inoperable Left Renal Vein Leiomyosarcoma Refractory to Chemotherapy Invades Inferior Vena Cava and Right Atrium: A Case Report

**DOI:** 10.7759/cureus.13182

**Published:** 2021-02-06

**Authors:** Geoffrey Lindblad, Stephanie Prater, Sotiris E Chaniotakis, James Banks

**Affiliations:** 1 Radiology, Aventura Hospital and Medical Center, Aventura, USA

**Keywords:** primary leiomyosarcoma, radiology, ct abdomen, radiation oncology, surgical oncology, mri abdomen

## Abstract

Mesenchymal stem cells differentiate into a plethora of cell types. These differentiated cell types include osteoblasts, chondrocytes, myocytes, and adipocytes. Sarcomas occur secondary to malignant transformation of these mesenchymal, pluripotent stem cells. Involuntary smooth muscle is responsible for the make up of hollow organs and vasculature in our body and is regulated by our autonomic nervous system, hormones, as well as chemical and local mediators. Cancer involving smooth muscle cells is designated as leiomyosarcoma (LMS). LMS can arise from any location in the body where smooth muscle is present and is frequently reported in the abdomen and pelvis, as well as the trunk, extremities, and throughout the retroperitoneum.

## Introduction

Sarcomas represent less than a fraction of a percent of overall malignancies [1]. They are part of broad group of neoplasms stemming from a mesenchymal origin with greater than 70 differentiating histological classifications. The two sub-groups of sarcomas include those of soft-tissue origin, and those originating from bone. In 2015, there was an estimation of more than 1.5 million new cancer cases reported in the United States. Of those diagnosed that year, nearly 12,000 were reported as soft-tissue sarcomas, and 3,000 as bone sarcomas [1]. Incidence is greater in adults than adolescents and children, with men being slightly more affected than women [1].

Leiomyosarcoma (LMS) occurs secondary to malignant transformation of smooth muscle cells in our body. The exact etiology of LMS is unknown, however, like many other cancers, genetics and environmental factors can likely be attributed. LMS is differentiated from its counterpart, leiomyoma, with radiologic and histopathologic evaluation (HPE). The presence of cellular atypia, high mitotic rate and areas of focal necrosis on HPE favor a diagnosis of LMS. On magnetic resonance imaging (MRI), LMS will often display nodular borders, inhomogenous enhancement with intralesional vessels and hemorrhage on T2-weighted images, with hypervascularization, peripheral early enhancement, and central necrosis [2].

According to an epidemiological study by the United States National Cancer Database from 2002-2014, the Surveillance, Epidemiology and End Results (SEER), found that of the 78,527 patients with a diagnosed sarcoma, 11,487 were LMS, making it the most commonly reported soft-tissue sarcoma diagnosed, followed by malignant fibrous histiocytoma (5,703), and liposarcoma (8,855). The top three reported primary tumor sites in this study included soft tissue (34,064), abdominal viscera (19,533), and thoracic viscera (2,588) [3]. LMS of intrinsic renal origin may destroy the renal parenchyma, whereas LMS of capsular origin may grow to surround the kidney [4]. These tumors have a tendency to invade the renal vein and subsequently the inferior vena cava (IVC), as seen in the presented case. In similar fashion to other sarcomas, LMS can spread throughout the bloodstream and can affect any soft tissue in the body. Sites of metastases reported most frequently to least include the lungs, peritoneum, liver, muscle, bones, and lymph nodes respectively [5].

Retroperitoneal sarcomas are commonly diagnosed late in the disease course as patients are often asymptomatic until the tumor begins obstructing nearby structures. According to studies at Memorial Sloan Kettering, New York, the five-year survival rate of patients with locally-advanced LMS at diagnosis is largely dependent on the ability to be surgically resected. With complete resection and clear margins, the five-survival rate approaches 60%. With partial resection or unclear margins, the five-year survival is roughly 35% [6].

We present a case of a 50-year-old female with a previously diagnosed LMS of the left renal vein that extended throughout the IVC eventually terminating in the right atrium who continued to have disease progression despite undergoing chemotherapy treatment.

## Case presentation

Our patient is a 50-year-old female that presented to our emergency department after suffering from a gradual onset of symptoms including dyspnea on exertion, left-upper quadrant abdominal pain, left-sided flank pain, and decreased appetite for a duration of two weeks.

Prior to arriving at our institution, our patient had a biopsy proven diagnosis of a left renal vein LMS made six months earlier and had since been undergoing chemotherapy treatment at a neighboring hospital. The patient finished an eight-week course of doxorubicin, gemcitabine, and docetaxel anhydrous two-weeks before presenting to us. An interval computed tomography (CT) scan of the chest, abdomen and pelvis was performed following this chemotherapy course which demonstrated a large thrombus in the IVC not appreciated on prior imaging. Staging at this point was documented as T3N0M0. The patient had been informed that there were no further chemotherapeutic options left available to her and so she began investigating surgical options which is when she established care with our own surgical oncologist. The patient notified our surgical oncologist of her new onset symptoms and it was recommended that she go the emergency department and be admitted for further evaluation due to the nature of her complaints in this context.

Vitals signs in the emergency department remained stable. The patient was afebrile with a temperature of 98.6 degrees Fahrenheit, a respiratory rate of 18 breaths per minute, a left-arm seated blood pressure of 120/65 mmHg, a pulse rate of 88 beats per minute, and bedside pulse oximetry of 98%. Complete cell counts (CBC), complete metabolic panels (CMP), coagulation profiles, and a urinalysis were obtained. Initial CBC was significant for a moderate anemia with a hemoglobin of 8.7 g/dL (12.0-16.0 g/dL) for which she was being treated with epoetin alpha. The CMP, coagulation profile and urinalysis were unremarkable. 

**Figure 1:**
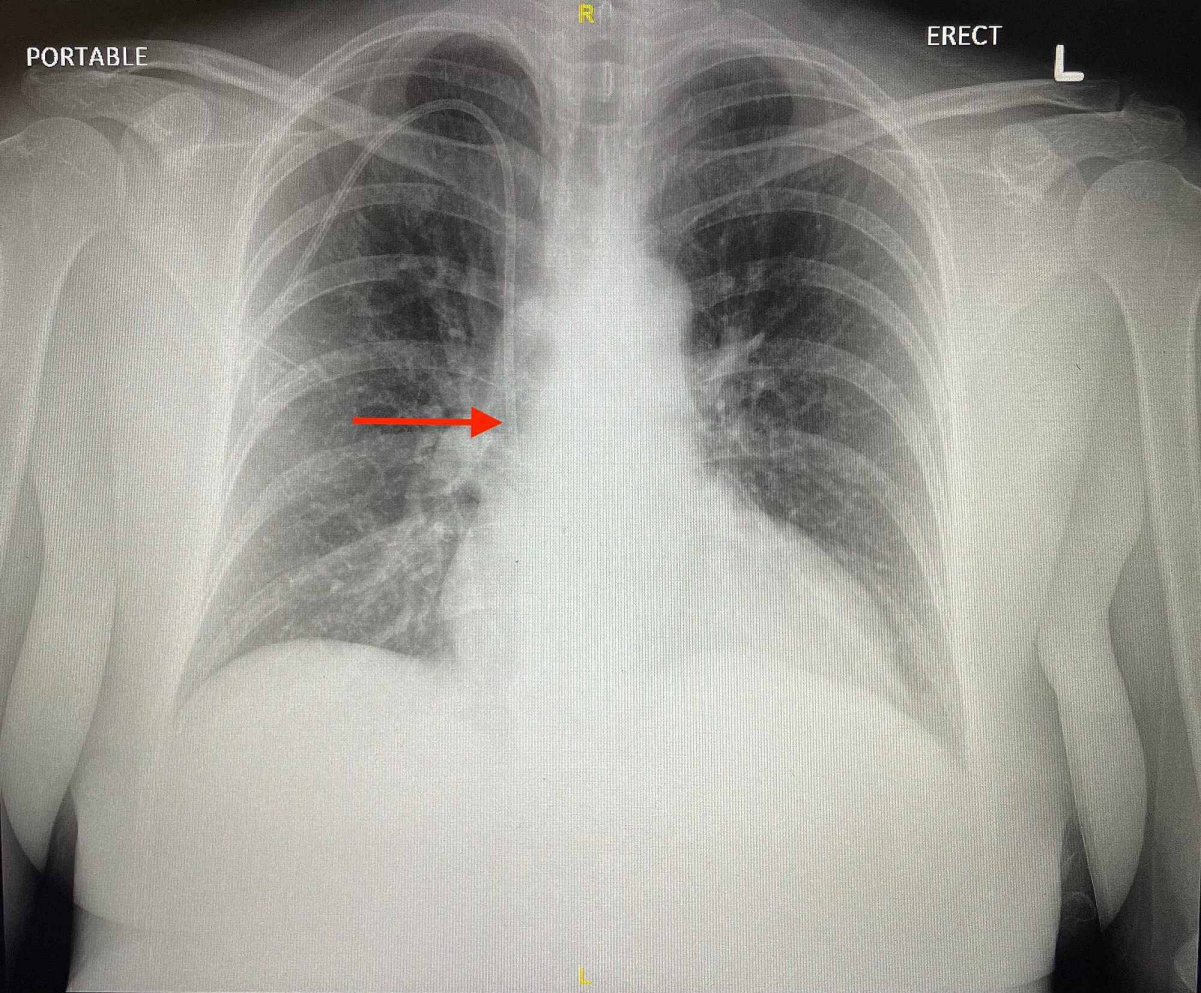
Chest radiograph showing a right-sided port-A-cath tip overlying the distal superior vena cava (red arrow)

During the hospital course, the patient underwent numerous imaging studies. Initial chest radiograph (Figure 1) showed no evidence of focal infectious consolidation, no pneumothorax, no pulmonary edema, and no significant pleural effusions. 

No segmental mismatched perfusion defects were identified in either lung on ventilation-perfusion scan, representing a very low probability for a pulmonary embolus. 

**Figure 2:**
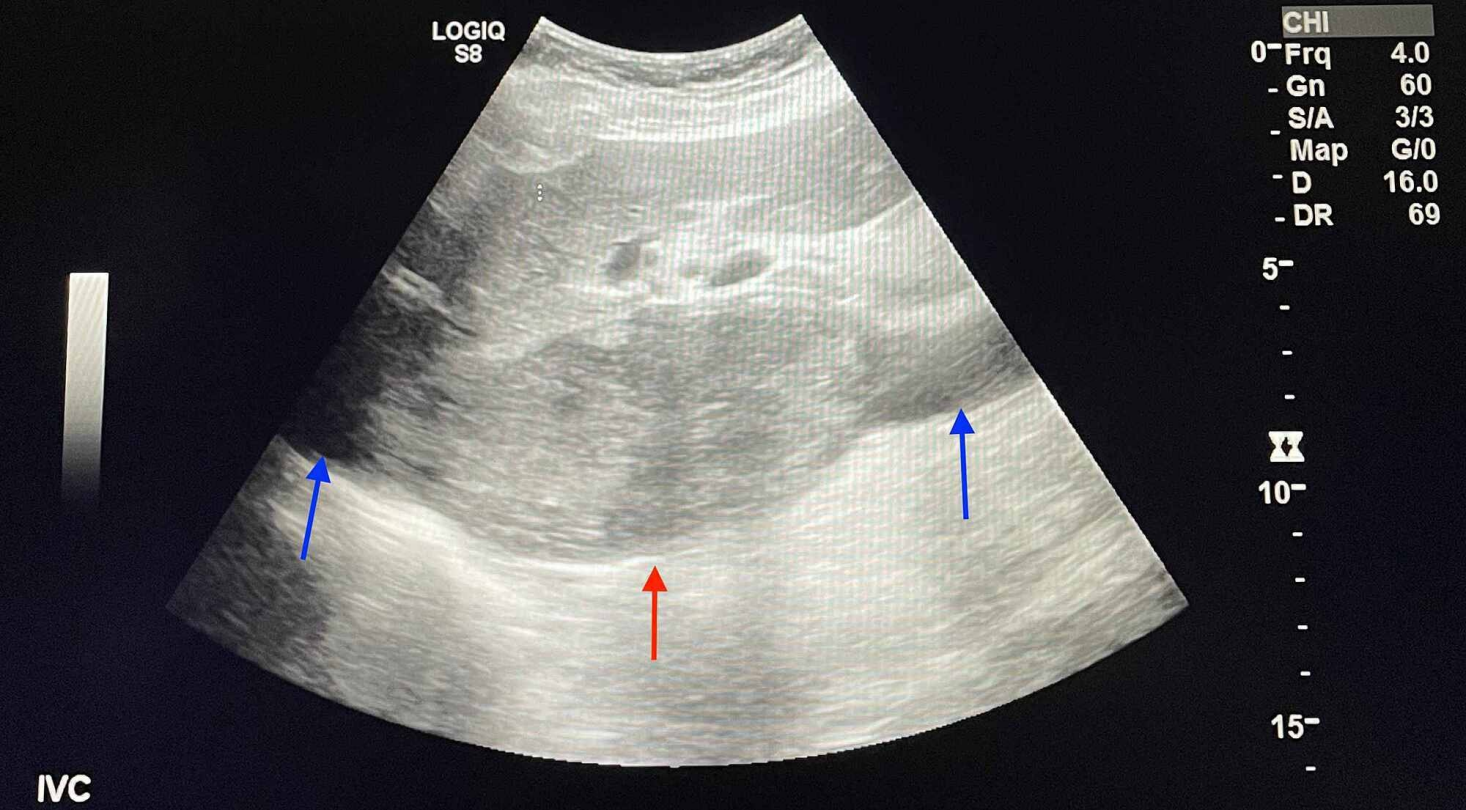
Ultrasonography with thrombus (red arrow) in proximal inferior vena cava (blue arrows)

**Figure 3:**
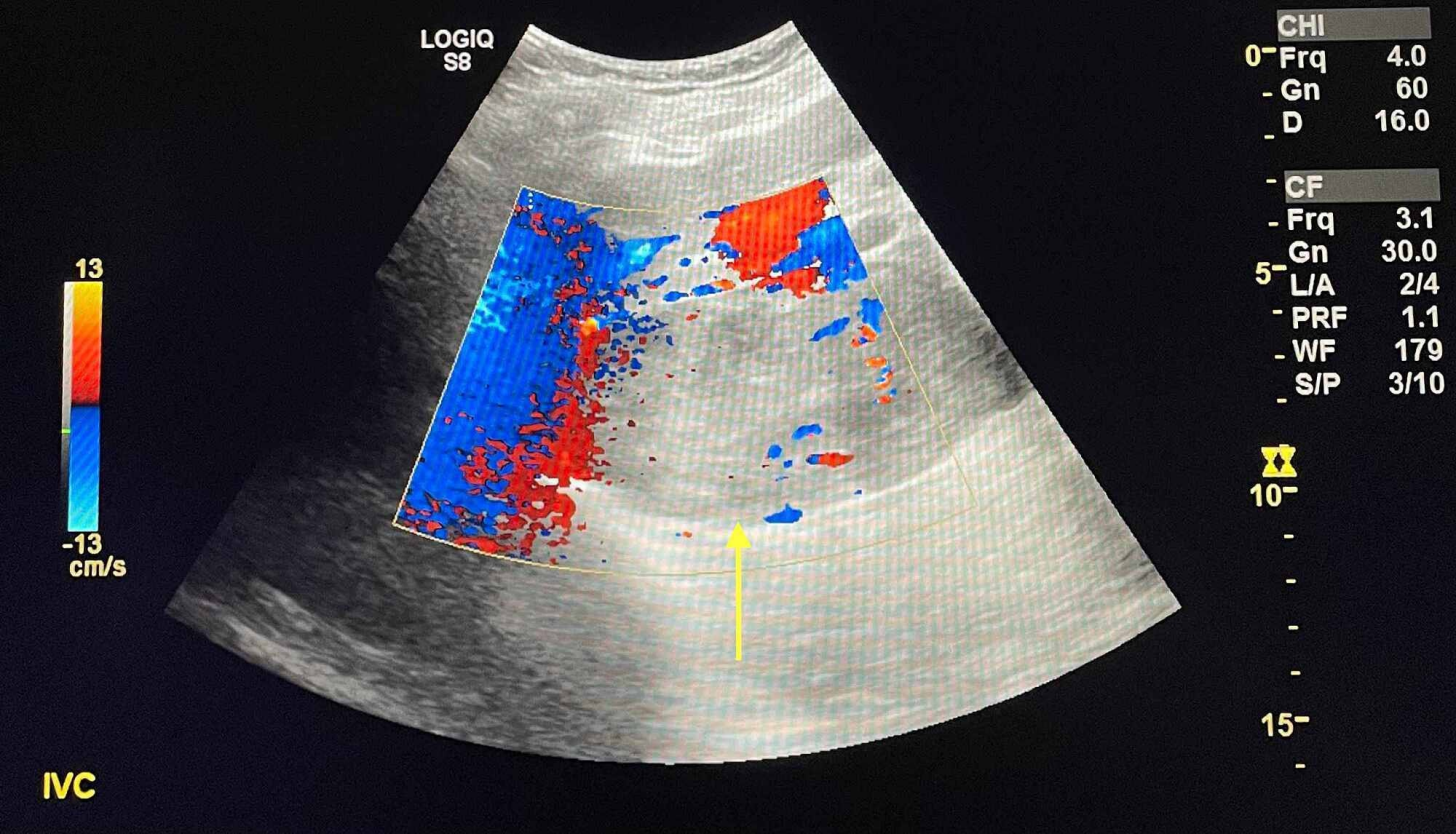
Doppler ultrasonography of proximal inferior vena cava with thrombus (yellow arrow) and decreased flow secondary to obstruction

Duplex ultrasound of the lower extremities resulted negative for deep vein thrombosis bilaterally. Right-upper quadrant (RUQ) doppler ultrasound was ordered to assess the liver, gallbladder, and IVC. An echogenic thrombus within the proximal IVC was seen (Figure 2, 3).

**Figure 4:**
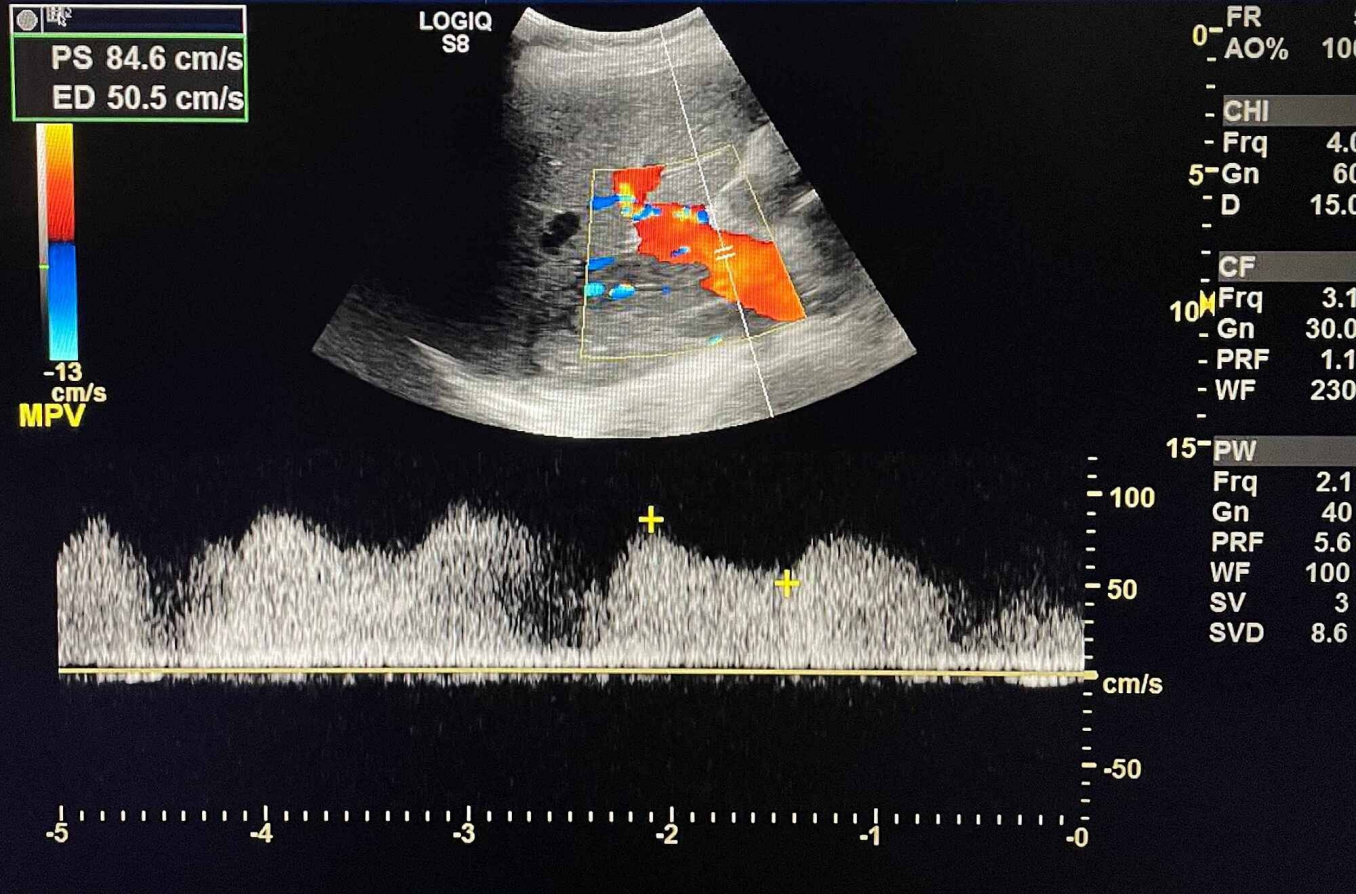
Doppler ultrasonography of liver showing hepatopetal, pulsatile flow in the main portal vein

In addition, the main portal vein displayed pulsatile flow (Figure 4), often seen in the setting of a right-sided cardiac dysfunction. 

A nuclear medicine triple-phase renal scintigraphy was also performed to assess the degree of renal involvement and functional status. The results showed evidence of increased flow and secretion in the right kidney when compared to the left. The left kidney demonstrated decreased excretion within the excretory range which can be indicative of mild obstruction versus tubular injury.

**Figure 5:**
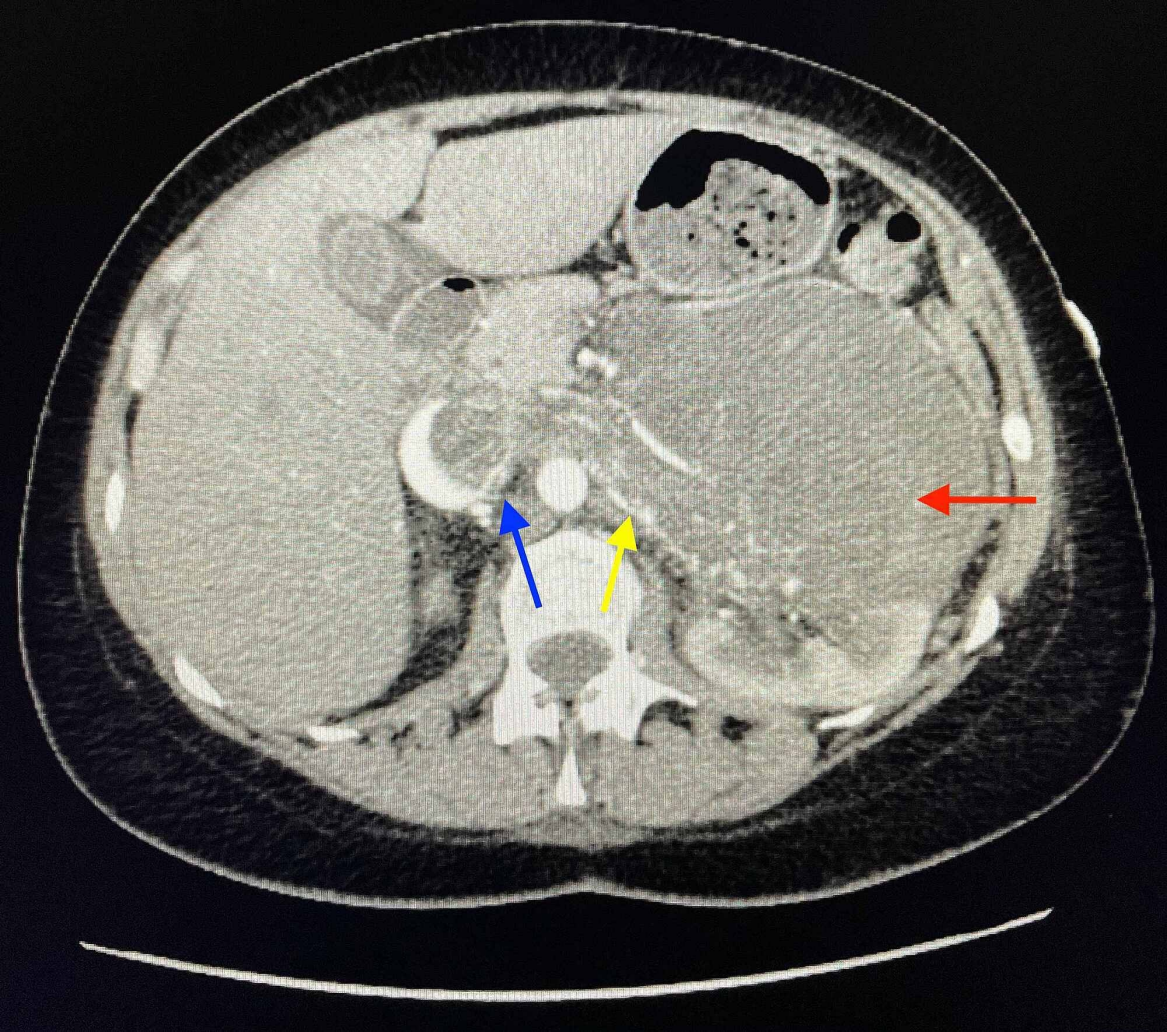
Axial-CT scan of abdomen with contrast showing a large, left-sided retroperitoneal mass arising from left renal vein (yellow arrow) with invasion of left kidney (red arrow) and inferior vena cava (blue arrow) CT: Computed Tomography

**Figure 6:**
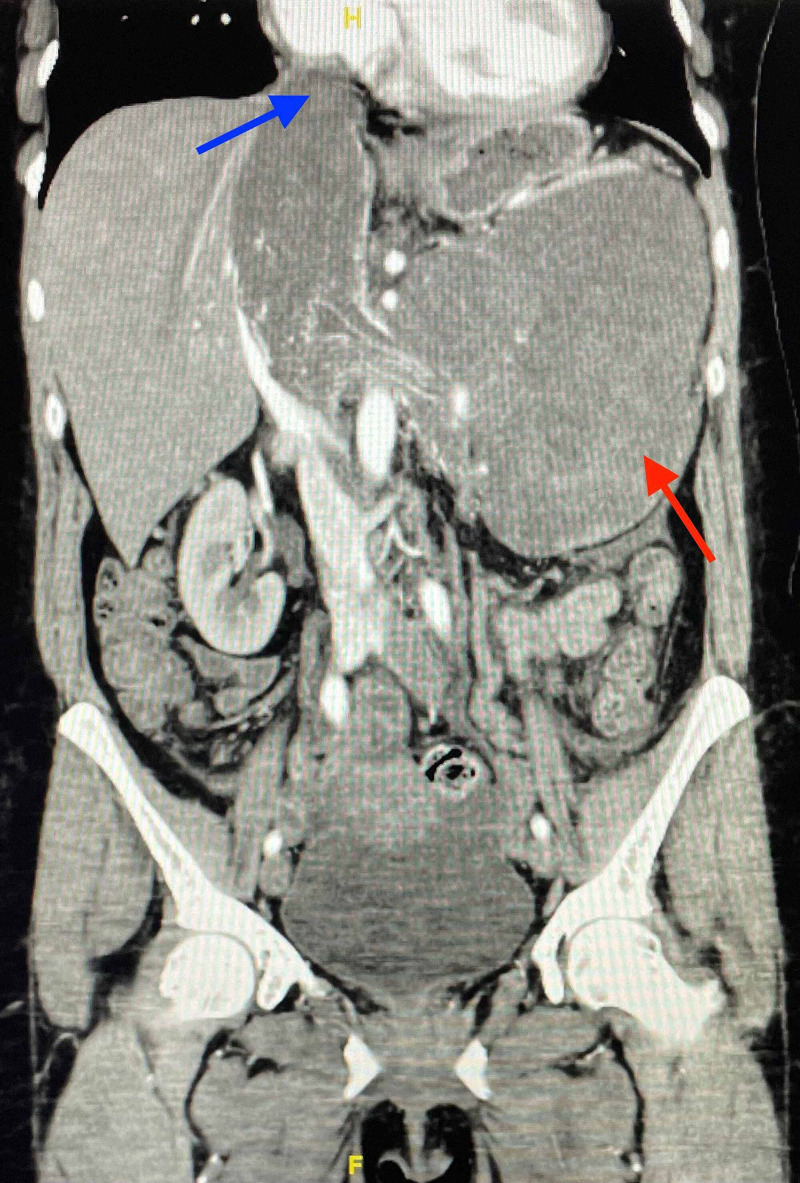
Coronal-CT scan of abdomen with contrast demonstrating a large, left-sided retroperitoneal mass (red arrow) arising from left renal vein with invasion of left kidney and inferior vena cava extending into the right atrium (blue arrow) CT: Computed Tomography

A CT scan of the chest with contrast showed no evidence of metastatic disease. Repeat CT scan of the abdomen and pelvis with contrast demonstrated a large inhomogenously enhancing left retroperitoneal mass arising from the left renal vein with invasion of the left kidney and accompanying intruluminal extension into the left gonadal vein, and IVC (Figure 5, 6). 

**Figure 7:**
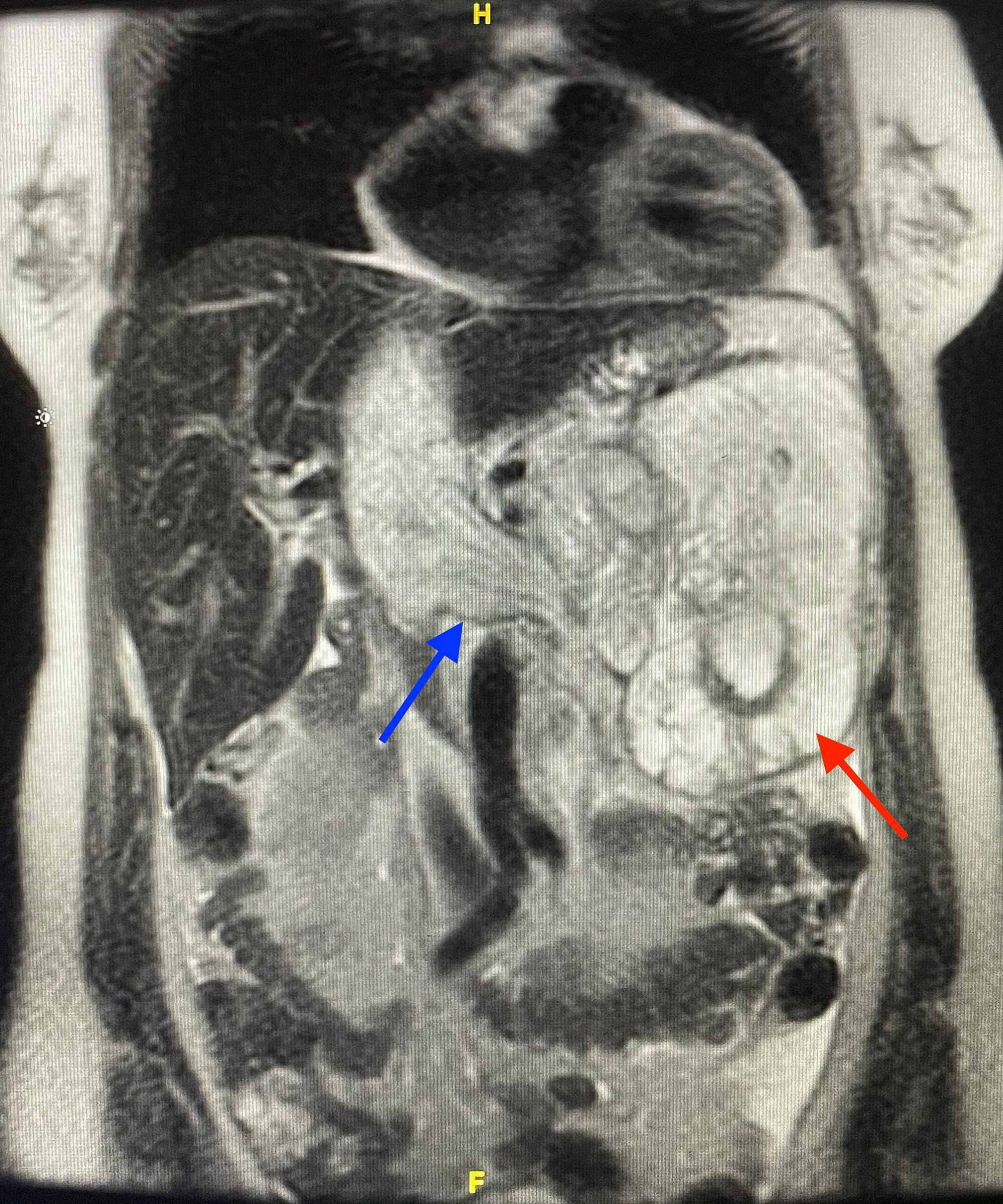
Coronal-T2-weighted MRI without contrast showing left-sided retroperitoneal mass (red arrow), extending across the midline into the inferior vena cava (blue arrow) MRI: Magnetic Resonance Imaging

To further evaluate the mass evident on CT abdomen with contrast, an MRI scan of the abdomen and pelvis with and without contrast was ordered. On the MRI, the mass measures 10.2 x 12.7 x 14.8 cm (transverse, anterior-posterior, cranio-caudal). The mass was characterized as T1 hypointense, T2 heterogenous and mostly hyperintense. The apparent epicenter is visualized at the left renal vein where the most avid area of enhancement is noted exerting a mass effect on the left kidney and invades the renal sinus venous structures (Figure 7). 

**Figure 8:**
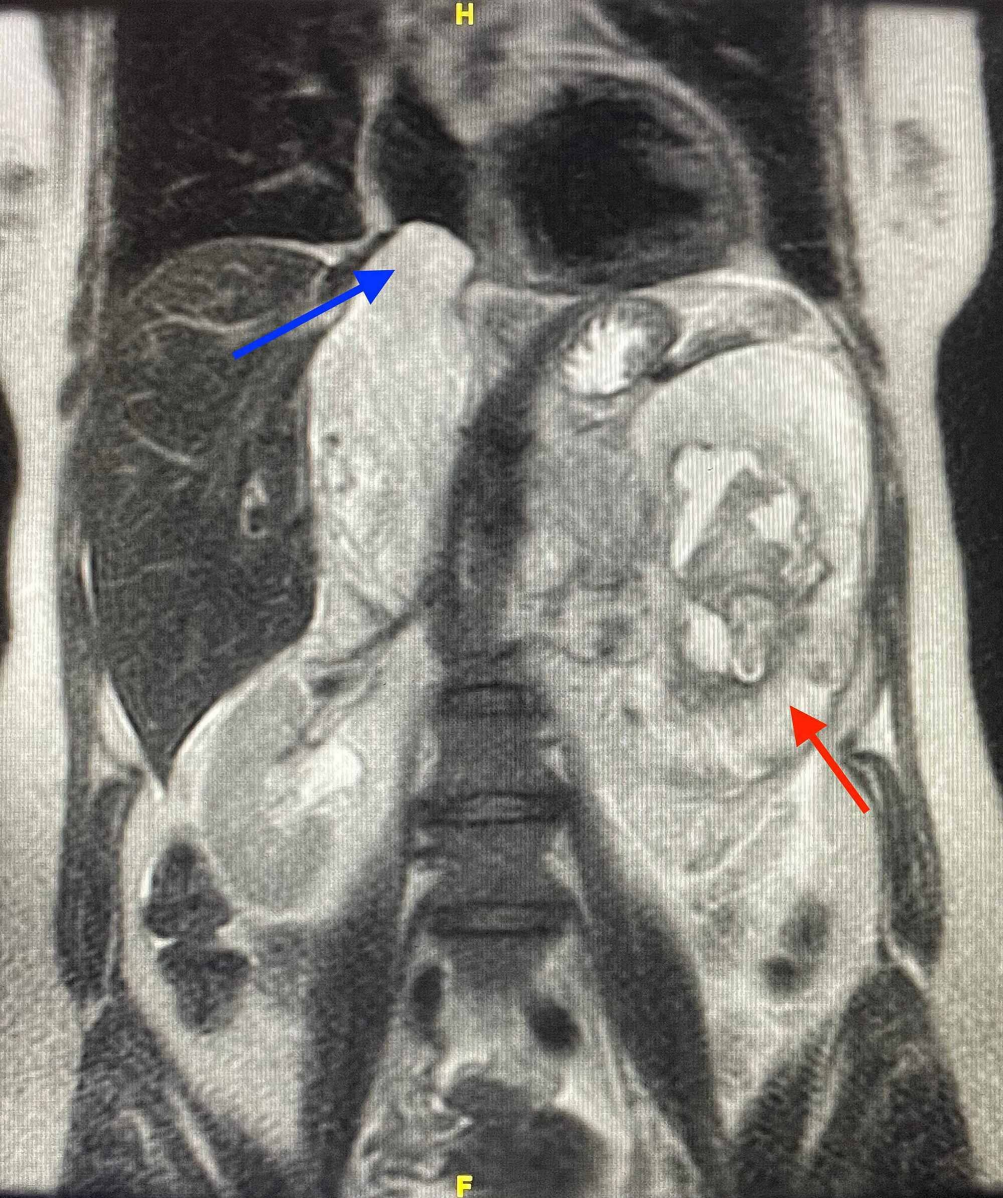
Coronal-T2-weighted MRI without contrast showing further extension off mass (red arrow) throughout the inferior vena cava expanding into the right atrium (blue arrow) MRI: Magnetic Resonance Imaging

The tumor extends intraluminally into the superior portion of the left gonadal vein and across the midline into the IVC, expanding into the right atrium by approximately 1.3 cm (Figure 8). 

Following laboratory and imaging work-ups, and evaluations by consulting specialists from Cardiology, Hematology, Pulmonology, Radiology, and Surgical-Oncology, our patient underwent a diagnostic exploratory laparoscopy as planned. Fortunately, the laparoscopy did not show evidence of gross metastatic disease. However, due to the size of the mass, proximity of vital organs and vessels, and tight communication with the IVC, surgical resection was not opted for at this time.

The following day, the patient was seen by Radiation-Oncology and findings from the laparoscopy were discussed. They recommended outpatient follow-up to determine candidacy for radiation therapy. The consulting specialists agreed the patient was stable from a respiratory and hemodynamic standpoint and able to be discharged to home on hospital day four as her symptoms subsided. Hematology-Oncology recommended a 30-day course of apixaban for anticoagulation. Outpatient follow-up with Radiation-Oncology, Surgical-Oncology, and Hematology-Oncology was advised and communicated with the patient and included in the discharge instructions. Unfortunately, the patient was lost to follow-up. 

## Discussion

The exact pathophysiology and the formation process of sarcomas are not well understood and less frequently described in literature. DNA alterations in tumor suppressor genes such as TP53 and ATRX chromatin remodeler have been identified in respect to their involvement in the process of LMS formation and mutational burden [7]. Further information gathered from a genetic standpoint may one day allow us to make predictive models of disease progression helping guide and tailor management. 

Most cases of sarcoma are idiopathic in nature, while a remaining subset are acquired, and can occur secondary to environmental factors. Environmental factors that have been linked to sarcoma development include ionizing radiation, and carcinogens seen in various herbicides, arsenic, polyvinyl chloride and more [8]. Due to the vast array of locations that a LMS can arise from, making a diagnosis heavily relies on imaging and histologic examination of biopsy specimens. Differential diagnoses for retroperitoneal LMS include, but are not limited to, undifferentiated pleomorphic sarcomas (formerly known as malignant fibrous histiocytoma), liposarcoma, and metastases. 

Histopathologic images from our patient were not able to be obtained for this case report, but LMS tend to display bundles of spindle-shaped cells, with flat-ended nuclei, in addition to eosiniphilic, fibrillary appearing cytoplasm. Those from the retroperitoneum show nuclear atypia with mitoses. Immunohistochemistry of LMS usually demonstrate positive staining for myogenic markers such as caldesmon, desmin, or smooth muscle actin (SMA) in greater than 70% of cases, and less frequently stain positive for cytokeratin and epithelial membrane antigen (EMA) in roughly 40% of cases [9].

Imaging findings of retroperitoneal LMS on CT and MRI scans are nonspecific. Similarly to other sarcomas on MRI, retroperitoneal LMS appear to have decreased signal on T1-weighted imaging and increased signal on T2-weighted imaging [10]. Although not ordered for our patient, magnetic resonance angiography (MRA) could have been potentially useful for surgical mapping due to the ability of the modality to display a multi-planar visualization of the intravascular components of the mass and its extending exterior vasculature. On RUQ ultrasound, our patient displayed hepatic venous outflow obstruction, also known as Budd-Chiari syndrome (BCS) secondary to the tumor thrombus in the IVC. The right, middle, and left hepatic veins remained patent, in addition to the intrahepatic IVC. However, CT findings commonly seen in BCS such as early enhancement of the caudate lobe and central liver surrounding the IVC, delayed peripheral enhancement with central low density known as the flip-flop appearance, and an inhomogenous mottled liver appearance were not evident. 

The prognosis and five-year survival rate of LMS strongly correlate with factors such as if the tumor can be surgically resected, tumor location, and if local extension and/or metastases are present. Surgical resection is known to be the standard of care for therapy in LMS. However, there is no consensus on standard radiation and chemotherapeutic regimens for retroperitoneal LMS. A case reported by Sharma et al. reported success using doxorubucin, ifosfamide, dacarbazine, and mesna combined with radiotherapy, however the renal LMS measured only 3.8 x 3.6 cm and was designated as low-grade [11], in contrast to the mass in our patient that measured three times the size. In another case report, Beccia et al. prescribed vincristine and cyclophosphamide with dactinomycin to a patient status post nephrectomy, which resulted in no metastasis at follow-up four years later [12]. Malik et al. reported similar results at one-year follow-up after the patient completed six cycles of adjuvant chemotherapy with daily doxorubucin and radiation [13]. On the contrary, our patient underwent a regimen of doxorubicin, gemcitabine, and docetaxel anhydrous without favorable results or significant improvement in disease burden.

No randomized control trials have demonstrated the efficacy of radiation and chemotherapy in locally-advanced or metastatic renal LMS cases, and therefore this topic warrants further investigation for patients who are not surgical candidates or continue to have disease progression while undergoing chemotherapy, as seen in our patient. However, for localized renal LMS cases, current experimental evidence suggests against the use of radiotherapy as a neodjuvant option. The STRASS I trial, an open-label, randomized, phase 3 study conducted on 266 patients with histologically documented, localized, primary retroperitoneal sarcoma demonstrated no survival benefit with neoadjuvant radiotherapy prior to surgery [14]. The STRASS II trial is currently underway and aims to investigate chemotherapy following surgical resection results versus surgical resection alone. 

The risks and benefits of adjuvant, neoadjuvant, and surgical therapy should be considered carefully in the context of each patient, as previous case reports have mentioned favorable outcomes, others have not. 

## Conclusions

Retroperitoneal LMS typically require surgical resection, chemotherapy, and in certain cases targeted radiation. Histologic and radiologic evaluation are vital aspects of the diagnostic workup and aid in creating individualized treatment plans. As previously mentioned, this malignancy is often diagnosed late in the disease course, when a patient experiences symptoms secondary to mass effect such as abdominal pain, cramping, and early satiety and/or loss of appetite. Our case demonstrates the need for multi-specialist care teams in order to give patients who are unfortunately diagnosed with a retroperitoneal LMS the best outcomes.
